# The Dynamic between Self-Efficacy and Emotional Exhaustion through Studyholism: Which Resources Could Be Helpful for University Students?

**DOI:** 10.3390/ijerph20156462

**Published:** 2023-07-27

**Authors:** Domenico Sanseverino, Danila Molinaro, Paola Spagnoli, Chiara Ghislieri

**Affiliations:** 1 Department of Psychology, University of Turin, 10124 Turin, Italy; domenico.sanseverino@unito.it (D.S.); chiara.ghislieri@unito.it (C.G.); 2 Department of Psychology, University of Campania “Luigi Vanvitelli”, 81100 Caserta, Italy; paola.spagnoli@unicampania.it

**Keywords:** academic self-efficacy, studyholism, emotional exhaustion, study load, teaching staff support, university students, COVID-19

## Abstract

While university students have experienced increased stress, anxiety, and study obsession (studyholism) during the COVID-19 emergency, supportive university environments and academic self-efficacy (ASE) were found to be protective factors. However, the perception of overstudying could have impaired ASE’s protection against studyholism, akin to workaholism. Following the job-demands resource model, this contribution examines the moderating roles of study load and teaching staff support in the relationship between ASE and exhaustion, mediated by studyholism. 6736 students from 11 universities (69.8% females; Mean age 24.67 years) completed a self-report survey concerning various academic and life aspects. Results showed that ASE was partially mediated by studyholism in its negative relationship with exhaustion. Both study load and support moderate this relationship, although the interaction effect between studyholism and ASE is positive. Nonetheless, ASE plays a protective role for all levels of study load and support, while studyholism is confirmed to have a significant impact on exhaustion, both directly and through its mediating role. Considering the high scores of both studyholism and exhaustion in this sample, the enhancement of ASE should be complemented by teacher support centered around opportunities to review study strategies with the students and strong attention to preventive measures, such as in itinerant evaluation, which could enhance both the perception of positive support and strengthen ASE.

## 1. Introduction

The COVID-19 pandemic has prompted scholars to shed light on academic life, which has been affected by significant changes due to social isolation, uncertainty about the job future, increased academic demands, and the introduction of Information and Communication Technologies (ICT) tools for distance learning. This context exacerbated an already difficult situation, considering that mental health issues were already prevalent among university students even prior to the pandemic, with the most common being anxiety disorders, mood disorders, and substance-related disorders [1]; furthermore, the need for mental health and well-being initiatives was already lacking before the pandemic [2]. In light of these concerns, several scholars have emphasized the importance of investigating university students’ well-being, showing increased stress, anxiety, loneliness, and depression [3,4,5,6,7,8] and lower levels of life satisfaction [9]. Other studies have highlighted an increased need for support [3] due to the excessive utilization of technology paired with an insufficient mastery of digital skills required for proficient remote learning, which could result in comparatively weaker academic achievements (e.g., lack of concentration and learning ability) [10,11]. As a result, university students reported reduced academic performance, with female students performing worse than their male peers [10,12]. Furthermore, the perception of increased academic workload was seen as an important source of distress (i.e., a feeling of frustration) [13], alongside increased anxieties regarding personal academic performance and financial circumstances [14,15]. On the other hand, Aucejo et al. [16] highlighted that 50% of the students in their research reported a decline in their study hours, 10% postponed their graduation or withdrew from their courses, and 40% of students lost their jobs. Conversely, some studies have found that personal resources (e.g., academic self-efficacy) and institutional resources (e.g., a supportive university environment) could exert a protective influence on the mental health of university students [17,18]. Arima et al. [19] have highlighted a negative relationship between academic self-efficacy and distress among Japanese medical students. Similarly, a longitudinal study by Reichel et al. [20] showed that academic self-efficacy plays a decisive role as a resilience-promoting factor among German university students since it is negatively related to emotional exhaustion, depression, and somatization. Regarding the Italian university context, a recent study by Ghislieri et al. [21] showed that self-efficacy represents a buffer resource for students’ well-being, especially against exhaustion.

In the same way, the academic community could represent a positive variable for university students, meaning that they feel part of a group and perceive support from it and its members (i.e., professors and peers). Indeed, as shown by Procentese et al. [22], a sense of responsibility and belonging to the academic community are negatively related to academic stress through self-efficacy.

It is worth noting that academic burnout is one of the leading research issues in the educational field nowadays, mainly because of its widespread presence over the years [23,24,25].

Conventionally, burnout is a state of chronic stress that comprises three dimensions: emotional exhaustion, cynicism and depersonalization, and a lack of professional accomplishment [26]. Similarly, concerning educational context, Schaufeli et al. [24] refer to student burnout as the feeling of being exhausted due to the study load, having a cynical and detached attitude towards studying and feeling ineffective as a student. Similarly to the workplace setting, academic burnout can seriously affect students’ psycho-physical health, leading to negative consequences such as poor academic performance, the intention to drop out of studies, and suicidal thoughts [24,27,28].

Therefore, it is crucial in the academic context to identify risk and protective factors related to burnout among university students.

Self-efficacy, defined as one’s belief in being capable of performing the necessary actions to achieve goals [29], might be an important protective factor against academic burnout [30,31]. Most of the previous literature has mainly addressed the relationship between self-efficacy and burnout among teachers [32,33,34]. Thus, given the complexity of the pandemic situation and its possible consequences in the academic context, it is essential to understand the underlying mechanism linking the aforementioned variables among university students. Specifically, in the present study, we hypothesize that studyholism (or study obsession), defined as a heavy investment in studying [35], mediates the relationship between academic self-efficacy and emotional exhaustion, one of the central dimensions of academic burnout. Furthermore, we aim to shed light on possible variables, as moderators, that could strengthen or buffer the relationship between academic self-efficacy and studyholism.

Based on the Job Demands and Resources model adapted for higher education students (JD-R model) [36,37], we posit that a contextual demand (i.e., study load) and a contextual resource (i.e., perceived support from teaching staff) moderate the relationship between academic self-efficacy and studyholism. Although academic self-efficacy might play a protective role against studyholism, we expect it to have less impact on studyholism when students experience high levels of study load, namely the amount and intensity of academic activities closely related to studying and exam preparation [21]. On the other hand, we hypothesize that the buffering effect of high perceived support from teaching staff (i.e., the perceived satisfaction related to professors and other teaching staff support) may strengthen the protective role of academic self-efficacy on studyholism.

The present study aims to fill a gap in the literature by testing a moderated mediation model: study load and perceived support from teaching staff moderate the relationship between academic self-efficacy and studyholism. At the same time, the latter mediates the relationship between self-efficacy and emotional exhaustion. From a practical perspective, our results would be helpful for Universities to plan preventive measures against students’ emotional exhaustion by focusing on positive resources (i.e., strengthening the perception of teacher support and academic self-efficacy) and limiting adverse demands (i.e., study load and overstudy climate).

### 1.1. The Relationship between Academic Self-Efficacy, Studyholism and Emotional Exhaustion

According to social cognitive theory (SCT) [38], self-efficacy represents the ability of individuals to plan and act to achieve specific performance, thus influencing their goals, behavior, and values. In the last few decades, scholars have explained the burnout phenomenon through the lens of self-efficacy theory [39]: burnout, as Leiter [40] pointed out, is the result of a self-efficacy crisis. In support of this argument, previous studies have shown that among students, a higher level of self-efficacy is related to a lower level of burnout [25,30].

Thus, from this perspective, self-efficacy could be seen as a personal resource for university students to cope with stressors, protecting them from experiencing emotional exhaustion resulting from study demands [24,41]. Given that academic self-efficacy was a positive variable in students’ well-being during the pandemic [17], investigating the relationship between academic self-efficacy and emotional exhaustion among university students is of great relevance. In this context, Loscalzo and Giannini [42] have also highlighted the widespread phenomenon of studyholism (or study obsession) among Italian university students during the COVID-19 outbreak. Studyholism has been conceptualized, in line with Loscalzo and Giannini’s comprehensive model of workaholism [43] and Snir and Harpaz’s heavy investment model [44], as a possible new clinical condition characterized by obsessive-compulsive symptoms and low or high study engagement (e.g., disengaged studyholics or engaged studyholics).

The present study is based on Bakker and Demerouti’s [45] job demands and resources (JD-R) theory, which points out how specific job demands and resources can influence well-being in the work context. Job demands refer to physical, psychological, social, or organizational features of work that involve skills and effort and are therefore associated with physiological and/or psychological costs. In contrast, job resources refer to physical, psychological, social, or organizational aspects of work that can be functional in achieving work goals, reducing job demands and associated costs, and promoting personal growth and self-development [45]. Furthermore, the JD-R model is based on two different underlying psychological processes that play a crucial role in determining job strain and motivation (i.e., the health impairment process and the motivational process) [45]. Focusing on the health impairment process, employees with low levels of self-efficacy (conceived as a personal resource) perceive themselves to be unable to effectively control the work environment and cope with job demands (e.g., workload). This condition may lead to the depletion of employees’ mental and physical resources (i.e., exhaustion) and, in the long run, to health problems [46,47]. On the other hand, several studies have shown that self-efficacy is positively related to workaholism [48,49]. Interestingly, Mazzetti et al. [50] showed that employees with higher self-efficacy report greater levels of workaholism in a climate of overwork.

Drawing on these findings, we could speculate that academic self-efficacy may be positively related to studyholism. However, although workaholism and studyholism share common features [35], it must be emphasized that studyholism is conceived as an obsessive-compulsive (OCD)-related disorder. Since high self-efficacy leads to a reduction in OCD symptoms [51], and according to the JD-R model, it could act as a personal resource to better face some demands that could potentially foster studyholism, we hypothesize that academic self-efficacy is negatively related to studyholism. Furthermore, Loscalzo and Giannini [52] have pointed out that studyholism results in negative psychological consequences among university students, such as negative affect and general stress. Therefore, in the current study, we consider studyholism as a negative factor through which we could better explain the relationship between academic self-efficacy and emotional exhaustion.

**H1.**  *There is a negative direct association between academic self-efficacy and emotional exhaustion.*

**H2.**  *There is a negative indirect association between academic self-efficacy and emotional exhaustion through studyholism: academic self-efficacy is negatively related to studyholism, which in turn is positively related to emotional exhaustion.*

### 1.2. The Moderating Role of Study Load and Perceived Support from Teaching Staff in the Relationship between Academic Self-Efficacy and Studyholism

Given the above considerations, the JD-R model is suitable for the academic context, considered an ‘organisational environment’ for students. In this respect, students’ well-being results from the interplay of two main aspects: academic demands (e.g., study load and technological demands) and academic resources (e.g., self-efficacy) [53]. In support of this argument, Naylor [37] has examined the key factors influencing distress (e.g., depression, anxiety, stress, and burnout) among university students during the pandemic, using a combination of self-determination theory (SDT) and the job demands-resources (JD-R) model.

In the present study, we investigate the moderating effect of an academic demand (i.e., study load) and an academic resource (i.e., perceived support from teaching staff) on the relationship between academic self-efficacy and studyholism. Therefore, we will be able to gain a more detailed understanding of the aforementioned relationship, which in turn could lead to negative outcomes for student well-being (e.g., emotional exhaustion). Similarly to workload, study load represents the quantity and intensity of academic activities that are mainly focused on studying and preparing for exams [21]. Recently, Tasso et al. [13] have highlighted that during the pandemic, students experienced frustration due to academic workload, resulting in stress and poor psycho-physical health. In light of their findings, we hypothesize that study load, like workload for workaholism [54], could enhance studyholism to the detriment of self-efficacy. On the other hand, social support (i.e., perceived support from supervisors, colleagues, family, and friends) is a crucial factor that helps individuals deal with stress and job demands [46]. In this regard, we posit that university students’ perceived support from teaching staff may represent a resource that, combined with high levels of self-efficacy, may result in lower levels of studyholism.

**H3.**  *The relationship between academic self-efficacy and studyholism is moderated by study load (H3a) and satisfaction with teaching staff support (H3b), with a stronger relationship when the study load is low and satisfaction with teaching staff is high.*

**Figure 1:**
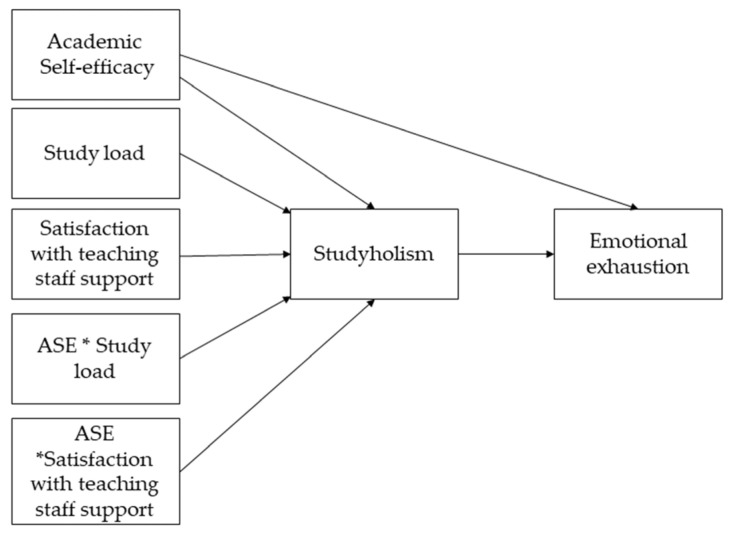
Theoretical model of the moderated mediation. The * represents the interaction term in the moderation analysis.

Figure 1 presents the theoretical model (without the control variables).

## 2. Materials and Methods

### 2.1. Procedure

This work was conducted within a more comprehensive research project of the National Conference for Equality in Italian Universities, an association of the University Committee of Guarantee that focuses its efforts on gender equality, work-life balance, welfare, inclusion, and integration in the Italian scholarly environment [21,55]. The study aimed to build an expansive database of the student population that can be used to compare the different academic contexts and design interventions and policies to improve students’ well-being. Invitations to participate in the investigation and descriptive support materials were delivered to all Committees of Guarantee associated with the network. Data was collected through a voluntary and anonymous online survey; each university was assigned a distinctive Limesurvey link. The university distributed the questionnaire independently through its communication channels, which were mainly the students’ institutional email accounts. The cover letter highlighted that participation was voluntary and anonymous, described the purpose of the study, and provided details about data handling. After data collection, each University could request their data to conduct targeted investigations; furthermore, the participating Committees could participate in a dedicated workshop where the aggregated data and its practical implications were discussed. The research was approved by the Bioethics Committee of the University of Turin on 30 April 2021 (Prot. No. 266199); the study was conducted in accordance with the Declaration of Helsinki [56], Italian data protection and privacy regulations (Law 196/2003), and General Data Protection Regulation (GDPR).

### 2.2. Participants

Participants filled out the online questionnaire between May and June 2021. Of the 10,298 students that completed the survey (a 4.69% response rate), this research considered only the 6736 cases that asked for teaching staff support regarding their study activities. Students were enrolled in 11 universities from Northern Italy (2 universities, 30.8%), Central Italy (3 universities, 6.1%), and Southern Italy and the Island (6 universities, 63.1%). Based on the number of enrolled students, Italian universities are categorized as small (up to 10,000 students; 2.1% of participants), medium (10,000 to 20,000 students; 16.9%), large (20,000 to 40,000 students; 59.9%), and mega (more than 40,000 students; 21.1%). Most respondents were women (69.3%), followed by men (30.0%), and people identifying as non-binary or in transition (0.7%). The mean age of the sample was 24.67 years (SD = 6.48), ranging from 18 to 68 years. Almost all participants were full-time students (91.6%); 57.9% of respondents were undergraduate students, followed by 26.9% of postgraduate students, and 15.2% of students were enrolled in a 5- or 6-year program, defined as a *ciclo unico*. 55% of students are enrolled in social sciences and humanities courses, with the most frequent areas being economics and statistics (13.1% of the total enrolled students) and antiquities, philology, literary studies, and art history (12.9%); the rest are enrolled in Science, Technology, Engineering e Mathematics (STEM) degrees, with medicine (14.4%) and engineering (14.2%) being the most common areas. Concerning academic success, some clarification is in order. In Italian Universities, there is no concept of failing a semester, and although classes can still be failed, students can retry them in order to acquire the necessary number of credits for each year. If students reach the end of a degree program without meeting the total number of credits required by the program, they can enroll for supplemental years; these students are designated as *fuori corso* (out of course). While only 15.7% reported that they are taking supplemental years to complete their courses, 50.3% of the participants stated that they are further behind on their exam schedule than they expected, 35.4% stated that they are on par with their exams, and only 14.4% declared that they are further beyond what they expected. The majority of students are not employed (69.6%), followed by part-time (17.7%) and full-time (8.3%) workers; finally, 1.6% of students were temporarily employed in paid university activities. Less than half of the respondents are residential students (43.3%).

We asked participants to rate their concerns about contracting COVID-19 in everyday life and finding a secure job on a scale from 1 (not at all) to 5 (very much).

The mean score for COVID concerns was 3.36 (SD = 1.24), while the mean score for job security was 4.16 (SD = 1.14).

### 2.3. Measures

Academic self-efficacy (ASE) was measured with four ad hoc items on a scale ranging from 1 (“never”) to 5 (“always”), based on the general Self-efficacy Scale of Chen et al. [57] We opted to design a new scale since we needed a short instrument in Italian specifically tailored to academic students. Participants were asked to rate how often they experienced a sense of motivation and readiness to confront academic challenges and attain their objectives. An example item is “Do you feel that you have the right skills to pass your exams?” The other items related to the effort to reach academic objectives, the effective distribution of their course load relative to other obligations, and the utilization of successful strategies in the academic career. Cronbach’s alpha was 0.83.

Satisfaction with teaching staff support was assessed with one ad hoc item with the following formulation: “In the last 6 months, with reference to your studies, how would you evaluate the support received from the teaching staff of your course?”. Participants were asked to rate this statement on a scale ranging from 1 (“Completely dissatisfactory”) to 5 (“Completely satisfactory”). Participants also had the possibility to opt out of the question if they did not ask for support; as already stated, these cases were excluded from the analyses.

Study load was measured with three items adapted from Bakker and colleagues’ scale [58] by substituting every mention of work with an appropriate expression concerning studying. Respondents were asked to rate the items on a Likert scale from 1 (“never”) to 5 (“always”). An example item is “I have to study under pressure”. Cronbach’s alpha was 0.73.

Studyholism was assessed with five items from the Loscalzo and Giannini Studyholism Inventory SI-10 [59], specifically those pertaining to the Studyholism factor of the instrument, which measures study obsession and concerns even when not actively studying. Participants were asked to indicate their degree of agreement on a Likert scale from 1 (“Strongly disagree”) to 5 (“Strongly agree”). An example item is “I have study-related worries even when I am not studying”. Following the author’s indications [60], the filler item “Before exams, I can’t think about anything else other than preparing for the exams” was left out when calculating the mean score of the scale. Cronbach’s alpha for the scale was 0.90.

Emotional exhaustion was measured with four items from Demerouti and colleagues’ scale [60], adapted for the academic environment following the same procedure applied to the study load. Respondents rated the items on a scale ranging from 1 (“never”) to 5 (“always”), referring to the last six months. A sample item is “When I’m studying, I often feel emotionally drained”. Cronbach’s alpha was 0.73.

For all variables, participants were asked to refer to the last six months.

### 2.4. Data Analysis

We conducted descriptive and correlational analyses in order to assess the characteristics of the sample. To ensure the reliability of the scales, we calculated Cronbach’s Alpha. Additionally, we performed an exploratory factor analysis to test the unidimensional structure of academic self-efficacy, using Unweighted Least Squares as an extraction method. We chose this method since the items were not normally distributed.

Once reliability was ensured, we calculated the mean score for each scale included in the analyses. We conducted a first-stage moderated mediation in order to assess whether there is an indirect effect of ASE on emotional exhaustion through studyholism and to investigate the possible moderating effects of study load and teaching staff support in the relationship between ASE and studyholism. We used Hayes’ [61] approach for dealing with more than one moderator in a mediated relationship, which entails performing two different ordinary least squares regressions. As is standard with PROCESS, missing data were handled with a listwise deletion. Moderation of the direct effect of the independent variable (in our case, ASE) was excluded, as it is optional. Every variable that defined a product was mean-centered prior to the analysis. In accordance with Hayes’ approach, we performed a bootstrapping procedure by extracting 5000 new samples from the original to estimate direct and indirect effects and confidence intervals.

All analyses were conducted using IBM SPSS, version 26 (IBM: Armonk, NY, USA, 2019), and the model 9 PROCESS for SPSS.

## 3. Results

Table 1 reports the means, standard deviations, and correlations of the study variables. The mean scores for study load, studyholism, exhaustion, and academic self-efficacy are all above the middle point of the scale, although the latter show the lowest among the listed variables. On the other hand, the mean score of satisfaction with teacher support is lower than the middle point, although the standard deviation is quite large (1.29). The correlations for the study variable were all significant for *p* < 0.01 in the expected directions: the resources are positively related between them and negatively related to studyholism and exhaustion, while the opposite is true for demands. Concerning the control variables, age is significantly correlated with every other variable for *p* < 0.01: positively with supplemental years, ASE, satisfaction with teaching staff support, and negatively with study load, studyholism, and exhaustion. Gender has significant negative correlations with supplemental years and ASE and positive correlations with all the other variables, although there is no significant correlation with satisfaction with support. Finally, being in the supplemental years of the course has negative correlations with ASE and satisfaction with teacher support and positive correlations with all other variables.

Since we used a brief, new scale to measure academic self-efficacy, we performed an exploratory factor analysis to test the unidimensionality of the instrument. First of all, we checked the univariate normality of the items, which was not supported, as shown in Table 2, which also reports the full formulation of the items.

We then proceeded to conduct the Exploratory Factor Analysis (EFA) using the unweighted least squares extraction, first by asking for a minimum eigenvalue of 1, and then by forcing the extraction of two factors. The final choice was a one-factor solution since, when trying to extract two factors, the Hessian matrix resulted in a negative definite. This is also in line with the rule of thumb stating that a factor should be defined by at least three variables with decent loadings. The one-factor solution explains 66.94% of the variance with 0% non-redundant residuals with absolute values higher than 0.05. Table 3 reports the structure matrix; all loadings were acceptable.

We tested our hypotheses by running a moderated mediation model in PROCESS for SPSS (model 9), controlling for age, gender, and being on supplemental course years. All predictors and control variables included were significant for *p* < 0.001. Study load (b = 0.50) and the interaction effect with ASE (b = 0.11) showed a positive relationship with studyholism. In contrast, ASE (b = −0.32), satisfaction with support (b = −0.07), and their interaction effects (b = −0.03) showed a negative relationship with the dependent variable. Being female (b = 0.22) and in supplemental years (0.15) both showed a positive relationship, while age has a negative relationship with studyholism. The model predicts 40.7% of the variance of the dependent variable. Table 4 presents the multiple regression with studyholism as the dependent variable.

**Figure 2:**
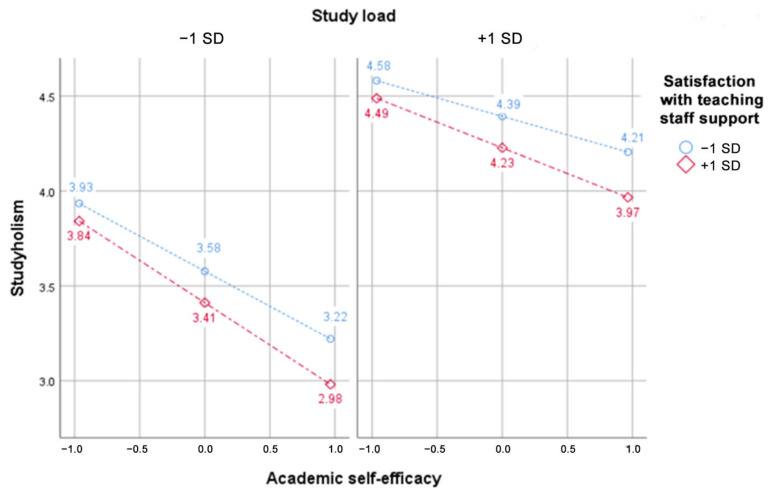
Moderation effects of study load and satisfaction with teaching staff support on the relationship between Academic self-efficacy and studyholism.

Table 5 reports the conditional effects of ASE on studyholism. While the coefficients for ASE are always greater when support is at +1 SD, it is apparent that the perceived level of study load played a larger role in determining the conditional effects of the focal predictor. Predictably, when support was at +1 SD and study load at −1 SD, ASE had the strongest negative relationship with studyholism, as can be seen in Figure 2.

It has to be noted that in this sample, since the mean score for study load was quite high (4.03), −1 SD does not represent low study loads but rather a fairly average amount of academic work.

The second regression is reported in Table 6. All effects were significant for *p* < 0.001, except for gender. ASE showed a negative relationship with exhaustion (b = −0.26), confirming a partial mediation through studyholism, which has a positive relationship with the dependent variable (b = 0.43). In this instance, both age (b = −0.19) and being in supplemental years (b = −0.11) showed a negative relationship with exhaustion. The model explains 47.1% of the variance in emotional exhaustion in this sample.

**Figure 3:**
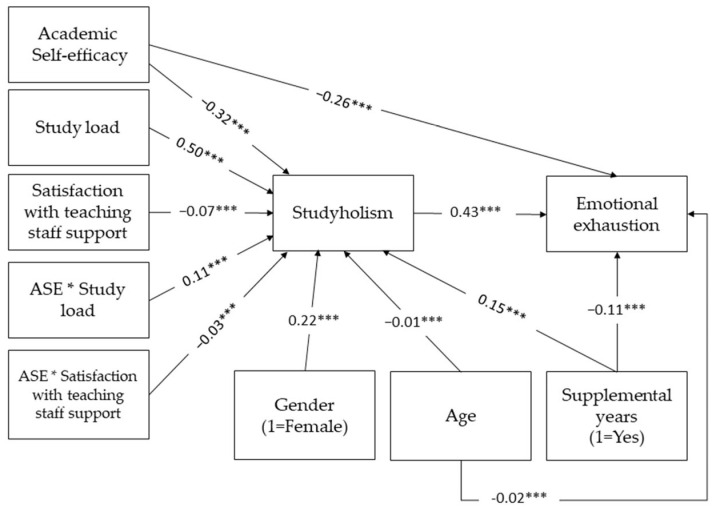
Moderated mediation model controlling for age, gender, and supplemental years. *** *p* < 0.001. Non-significant relationships not reported in the figure. The * represents the interaction term in the moderation analysis.

The bootstrapped conditional indirect effects (Table 7) and thus the total effects, similarly to the conditional effects in the first step, are stronger for +1 SD satisfaction with teaching staff support and −1 SD study load. Table 8 reports the indices of partial moderated mediation, which quantify the indirect effect of each interaction on emotional exhaustion when the other moderator is held constant. Both indices support the overall model (shown in Figure 3), as zero is not included in either of the 95% confidence intervals, although caution should be exercised concerning the second index considering its proximity to zero.

Everything considered, the results confirmed the hypotheses: ASE had a negative relationship with studyholism, and while the latter had a positive relationship with emotional exhaustion, the mediation is partial, and the indirect effect of ASE on exhaustion is negative for all conditions of the two moderators.

## 4. Discussion

Our results confirm that ASE could be considered an excellent protective factor against emotional exhaustion, a result in line with studies conducted before and during the COVID-19 pandemic [17,41]. However, the results of the moderation could shed further light on the interactions between personal and contextual resources and demands in determining when self-efficacy could potentially become more detrimental to students well-being.

Concerning Hypothesis 1, the results are in line with the JD-R model (and its health impairment process) and the conceptualization of studyholism as an OCD disorder [35] associated with a wide range of negative psychological consequences among university students, demonstrating the potential role of ASE as a personal resource that can reduce such negative outcomes. Further, this result supports previous studies showing that academic self-efficacy could represent a resilience-promoting factor against students’ burnout levels and emotional exhaustion [20,25,30].

In addition, studyholism had a positive relationship with exhaustion, with a considerably larger effect compared to the other variables; this also confirms hypothesis 2, since even if studyholism appears to have a strong role in determining emotional exhaustion, it only acted as a partial mediator between ASE and exhaustion, which is reflected in the negative direct and indirect effects; this result is also in line with the protective role of high self-efficacy concerning OCD symptoms [51].

From a theoretical point of view, these results confirmed the suitability of the JD-R model and its health impairment process in the school context, as highlighted by Naylor et al. [37] (i.e., among college students in the present study).

Hypothesis 3 was also confirmed. According to the JD-R model, ASE, intended as a resource, had more salience when satisfaction with teaching staff support, another contextual resource, was also higher. On the other hand, we did not find a greater positive effect of ASE in the presence of high demands. This could be explained in two ways: first, this proposition is valid when both demands and resources are high, and this was not the case for our sample, specifically for what concerns satisfaction from teacher support. Second, although studyholism and workaholism have some differences, they still share some aspects, and as already reported in the literature, workaholism stems from a combination of individual and contextual factors. Similarly, since students with high self-efficacy may perceive that they can study more and for longer, they could be more prone to problematic overstudying, especially when this behavior is rewarded. Concerning the control variables, we can see that being female has a positive relationship with studyholism. Once again, it is important to remark that we only measured the internalizing aspects of studyholism [35]. This could be partially accounted for by examining some personal antecedents of studyholism, namely personality traits. Neuroticism, conscientiousness, and perfectionism have been linked to both workaholism [62] and OCD [63], although Loscalzo and Giannini [52] found that perfectionistic strivings and concerns do not predict studyholism per se; again, the authors argue that this could be because these aspects can lead to the maladaptive facets of high study investment when students perceive a climate of overwork, which is the case in our study. Perfectionism and neuroticism have been shown to be linked, especially for women. More specifically, a meta-analysis by Smith et al. [64] found that perfectionistic concerns are characterized by neuroticism, low agreeableness, and low extraversion; perfectionistic strivings are characterized by conscientiousness; and that the positive links between perfectionism and neuroticism increased with the number of females involved in the analyses, especially concerning the association between self-oriented perfectionism.

The particular context of the COVID-19 pandemic may have played a crucial role. Social isolation could have prevented receiving positive feedback that sustains self-efficacy and can counteract some of the more harsh self-standards stemming from perfectionism. The increased study load accompanied by new learning challenges and the general sense of insecurity and anxiety could have led students, especially female students who are more frequently higher in neuroticism, conscientiousness, and perfectionism, to engage in overstudying as a maladaptive coping mechanism, not dissimilarly to what happened to perfectionists regarding workaholism [65]. In fact, Alsaady et al. [66] reported that female students had higher levels of exam anxiety, which can also be considered an antecedent of studyholism (i.e., social anxiety) [67]. The absence of a significant relationship between emotional exhaustion and gender may be explained by the inclusion of studyholism in the model, which could possibly act as a mediator between personal antecedents other than self-efficacy and emotional exhaustion. Going over the control variables, being in a supplemental course year was positively associated with studyholism, while age showed a negative relationship. The first relationship could be explained by rumination over one’s own academic future due to falling behind with exams. This may lead to the perception that previous efforts are insufficient to meet academic challenges, fostering an obsession with studying. This association is likely stronger among individuals who already impose rigid, perfectionist standards on themselves than among those aiming for realistic success. Loscalzo and Giannini’s [35] findings support this proposition, highlighting that trait anxiety and insecurity about the future are strong predictors of studyholism alongside other internalizing symptoms.

Interpreting the association between age and studyholism is more complex. Although students in supplemental years are typically older, the correlation between these variables in our study was weak. Older students may possess more refined coping strategies or a better understanding of their studying strategies, allowing them to manage their study load and anxiety more effectively. This could also explain the negative relationship between age and emotional exhaustion. However, we cannot definitively determine if older students had the opportunity to build these resources prior to the pandemic, while their younger peers may have been asked not only to face academic challenges for the first time but also during a global, unforeseen health emergency with limited access to resources and opportunities.

### Limitations

This research presents some limitations. First, it is a cross-sectional study, which prevents us from establishing causality; second, in order to make it easier for participants to complete the questionnaire, we often used shortened versions of validated scales, some of which were modified to fit the academic context. This also meant that when deciding how to assess studyholism, only the internalizing part was considered. Additionally, satisfaction with teaching staff support was assessed with an ad hoc measure. While these adjustments were made after thoughtful deliberation among the research team, it would be advisable for future investigations to use brief and meticulously validated assessments. This will ensure that the survey can be replicated systematically across various university settings and timeframes. Finally, although the sample size is large, with participants coming from different universities in terms of size, geographical location, and resources, future research could improve the response rate by devising more effective ways to engage the student population.

## 5. Conclusions

This study, although within the limitations of a cross-sectional survey, allowed for an in-depth investigation of the link between study load, a personal resource such as ASE, and a contextual resource such as teaching staff support, studyholism, and exhaustion in a large sample of Italian university students during the second lockdown to contain the COVID-19 Pandemic. Outside of the pandemic experience, the results are of interest in reflecting on the sustainability of academic life and identifying attitudes and actions that can counteract excessive and compulsive study, which is associated with negative consequences and therefore should be discouraged [35].

In this study, we noted the teaching staff’s support in a concise way, and qualitative insights would be needed to better understand how this suggestion might translate into operational directions. Looking at the relationship with ASE, however, we can already identify some directions. Support does not amount to facilitation or to some forms of consolation and comfort; the role of faculty is complex but always geared toward promoting the achievement of meaningful learning in the context of higher education. In this sense, consistent with the literature about soft skills [68], the results suggest the importance of providing students with protected opportunities prior to the assessment moment in which to test their knowledge and know-how and receive appropriate feedback with high formative value. In the early days of the pandemic, it might have been difficult to use technologies learned “in medias res,” for such a complex task as assessing students’ growth.

Today, outside of mandatory remote teaching, on the back of realized learning, this guidance can be translated into concrete behaviors that can become an integral part of teaching practice. University teaching staff can help support self-efficacy and reduce the presence and effect of studyholism in many ways. First, by representing a role model, it is important that the learning objectives defined are consistent with the actions and ways in which the teaching role is acted out, for instance by not rewarding excessive studying and actively monitoring their course load, both actions that can play a part in avoiding the association between success and overstudying. Some other aspects are, among others, particularly relevant: a shared passion for the discipline, in a non-obsessive but harmonious sense, to use Vallerand’s well-known distinction [69]; clarity in the definition of learning objectives; the definition of a clear formative pact; the search for balance in the proposal of the study load in relation to the formative objective, considering not only one’s own discipline but also the overall load of the study period; and the willingness to also provide, where possible and necessary, intermediate feedback, both to help “take stock” and to specify the expected level of preparation.

In this sense, it is important for universities to support teaching, both by allowing teaching staff to devote the proper amount of time to teaching activities to prepare for and accompany learning, reducing the burden of institutional and bureaucratic workload [70], and by providing opportunities for teaching training and support, including psychological support, where staff require it, because at a time of fatigue, it is difficult to live harmoniously in the teaching role. At the same time, in more general terms, awareness-raising campaigns against excessive and compulsive studying, aimed at both teaching staff and students, are useful in order to deconstruct the too-often positive image associated with those who “kill themselves studying”. This can be done through training moments aimed at highlighting dysfunctional dynamics but also through individual interventions, where necessary, in this case on voluntary motivation.

## Figures and Tables

**Table 1:** Means, standard deviations, and correlation coefficients of the study variables.

	M	SD	1	2	3	4	5	6	7	8
1. Age	24.67	6.48								
2.Gender (1 = Woman)	-	-	0.00							
3.Supplemental years	-	-	0.20 **	−0.03 *						
4. ASE	3.20	0.97	0.14 **	−0.03 **	−0.130 **	***0.83***				
5. Study load	4.03	0.82	−0.13 **	0.07 **	0.031 *	−0.30 **	***0.73***			
6.Satisfaction with teaching staff support	2.77	1.29	0.21 **	0.00	−0.040 **	0.41 **	−0.26 **	-		
7. Studyholism	3.86	1.07	−0.20 **	0.13 **	0.084 **	−0.46 **	0.52 **	−0.33 **	***0.90***	
8. Emotional exhaustion	3.85	0.95	−0.27 **	0.09 **	0.01	−0.50 **	0.46 **	−0.36 **	0.63 **	***0.73***

* *p* < 0.05 ** *p* < 0.01. Reliability coefficients are reported in the diagonal in bold italic. ASE = academic self-efficacy.

**Table 2:** Kolmogorov-Smirnov test of the four academic self-efficacy items.

	Statistics	*p*
1—Do you feel that you have the right skills to pass your exams?	0.203	<0.001
2—Do you feel motivated to attain your academic goals?	0.177	<0.001
3—Are you capable of efficiently managing your workload in relation to other commitments?	0.161	<0.001
4—Do you consider the strategies you have implemented to pursue your university career to be effective?	0.184	<0.001

**Table 3:** Structure matrix for the EFA of the 4 academic self-efficacy items.

	λ
1—Do you feel that you have the right skills to pass your exams?	0.695
2—Do you feel motivated to achieve your academic goals?	0.733
3—Are you capable of efficiently managing your workload in relation to other commitments?	0.745
4—Do you consider the strategies you have implemented to pursue your university career to be effective?	0.818

**Table 4:** Multiple regression with studyholism as dependent variable (*n* = 6446).

	b	t	*p*	LLCI	ULCI
Constant	4.08	92.07	<0.001	3.990	4.164
ASE	−0.32	−26.55	<0.001	−0.344	−0.296
Study load	0.50	37.22	<0.001	0.472	0.524
ASE * Study load	0.11	8.38	<0.001	0.082	0.131
Satisfaction with teaching staff support	−0.07	−7.23	<0.001	−0.082	−0.047
ASE * Support	−0.03	−3.53	<0.001	−0.046	−0.013
Age	−0.01	−8.57	<0.001	−0.018	−0.011
Gender (1 = Female)	0.22	10.07	<0.001	0.181	0.268
Supplemental years	0.15	5.09	<0.001	0.089	0.201

Dependent variable: Studyholism (Adjusted R^2^ = 0.406; *p* < 0.001). All variables that define products have been centred around the mean before analysis. LLCI = Lower limit confidence interval; ULCI = Upper limit confidence interval. ASE = academic self-efficacy. The * (as a multiplication term) represents the interaction term in the moderation analysis.

**Table 5:** Conditional effect of Academic self-efficacy on studyholism at ± 1 SD of the moderators.

	b	S.E.	LLCI	ULCI
Study load −1 SD, Support −1 SD	−0.37	0.021	−0.410	−0.329
Study load −1 SD, Support +1 SD	−0.45	0.018	−0.481	−0.409
Study load +1 SD, Support −1 SD	−0.20	0.017	−0.228	−0.161
Study load +1 SD, Support +1 SD	−0.27	0.021	−0.311	−0.230

S.E. = Standard Error.

**Table 6:** Multiple regression with exhaustion as dependent variable (*n* = 6446).

	b	t	*p*	LLCI	ULCI
Constant	2.64	48.94	<0.001	2.533	2.744
ASE	−0.26	−25.99	<0.001	−0.283	−0.243
Studyholism	0.43	46.38	<0.001	0.413	0.450
Age	−0.02	−13.24	<0.001	−0.210	−0.160
Gender (1 = Female)	0.03	1.77	0.077	−0.004	0.071
Supplemental years	−0.11	−4.41	<0.001	−0.154	−0.059

Dependent variable: Exhaustion (Adjusted R^2^ = 0.471; *p* < 0.001). ASE = academic self-efficacy.

**Table 7:** Bootstrapped conditional indirect effects and total effects of ASE on Exhaustion at ±1 SD of the moderators.

	b	Tot. Eff	Boot S.E.	BootLLCI	BootULCI
Study load −1 SD, Support −1 SD	−0.16	−0.42	0.011	−0.181	−0.138
Study load −1 SD, Support +1 SD	−0.19	−0.46	0.010	−0.212	−0.172
Study load +1 SD, Support −1 SD	−0.08	−0.35	0.007	−0.098	−0.070
Study load +1 SD, Support +1 SD	−0.12	−0.38	0.010	−0.136	−0.097

Boot S.E. = Bootstrapped standard error; BootLLCI = Bootstrapped Lower limit confidence interval; BootULCI = Bootstrapped Upper limit confidence interval.

**Table 8:** Bootstrapped indices of partial moderated mediation.

	Index	Boot S.E.	BootLLCI	BootULCI
ASE * Study load -> Studyholism -> Exhaustion	0.05	0.006	0.033	0.058
ASE * Support -> Studyholism-> Exhaustion	−0.01	0.004	−0.020	−0.005

The * (as a multiplication term) represents the interaction term in the moderation analysis.

## Data Availability

The data presented in this study are not publicly available due to Italian privacy law. The data are available on request from the corresponding author.
